# An assessment of emotional intelligence in emergency medicine resident physicians

**DOI:** 10.5116/ijme.5a2e.a8b4

**Published:** 2017-12-27

**Authors:** Dimitrios Papanagnou, Kathryn Linder, Anuj Shah, Kory Scott London, Shruti Chandra, Robin Naples

**Affiliations:** 1 Department of Emergency Medicine at Thomas Jefferson University, Philadelphia, Pennsylvania, USA; 2 Sidney Kimmel Medical College of Thomas Jefferson University, Philadelphia, Pennsylvania, USA

**Keywords:** Emotional intelligence, resident, wellness, graduate education, training

## Abstract

**Objectives:**

To define the emotional intelligence (EI) profile of emergency medicine (EM) residents, and identify resident EI strengths and weaknesses.

**Methods:**

First-, second-, and third-year residents (post-graduate years [PGY] 1, 2, and 3, respectively) of Thomas Jefferson University Hospital’s EM Program completed the Emotional Quotient Inventory (EQ-i 2.0), a validated instrument offered by Multi-Health Systems. Reported scores included total mean EI, 5 composite scores, and 15 subscales of EI. Scores are reported as means with 95% CIs. The unpaired, two-sample t-test was used to evaluate differences in means.

**Results:**

Thirty-five residents completed the assessment (response rate 97.2%). Scores were normed to the general population (mean 100, SD 15). Total mean EI for the cohort was 103 (95%CI,100-108). EI was higher in female (107) than male (101) residents. PGY-2s demonstrated the lowest mean EI (95) versus PGY-1s (104) and PGY-3s (110). The difference in PGY-3 EI (110; 95%CI,103-116) and PGY-1 EI (95, 95%CI,87-104) was statistically significant (unpaired t-test, p<0.01). Highest composite scores were in interpersonal skills (107; 95%CI,100-108) and stress management (105; 95%CI,101-109). Subscale cohort strengths included self-actualization (107); empathy (107); interpersonal relationships (106); impulse control (106); and stress tolerance (106). Lowest subscale score was in assertiveness (98). Self-regard (89), assertiveness (88), and independence (90) were areas in which PGY-2s attained relatively lower scores (unpaired t-test, p<0.05) compared to their peers and the general population. PGY-3’s scored highest in nearly all subscales.

**Conclusions:**

The EQ-i offers insight into training that may assist in developing EM residents, specifically in self-regard, assertiveness, and self-expression. Further study is required to ascertain if patterns in level of training are idiosyncratic or relate to the natural maturation of residents.

## Introduction

Emotional Intelligence (EI) has been broadly defined as one's ability to identify and manage his or her emotions and those of others.^1^ First recognized for its value in the business arena, EI has recently been applied to clinical medicine given its ties with academic and professional leadership, enhanced job performance, stress management, and emotional well-being.^2^ EI has been a cited key determinant promoting resilience among health professionals against burnout.^3^^,^^4^ Physicians who are better at expressing emotions had patients rate them as more caring, sensitive, and better at listening.^5^ Similarly and importantly in the era of physician metrics, patient satisfaction is strongly correlated to emotionally expressive nonverbal behaviors.^4^^-^^6^ Physicians are often faced with the challenge of perceiving and responding to their own emotions, as well as those of their patients and multidisciplinary teammates. Further understanding the internal thoughts and interlacing emotions a provider experiences is warranted, as it has the potential to improve patient care through enhanced communication and professionalism.^1^

The practice of emergency medicine is rooted in team-based health delivery. EM physician responsibilities include, but are not limited to, effectively communicating and properly empathizing with patients, families and their care teams; coordinating care with other providers; and expeditious, holistic decision making. Considering the degree to which EM physicians are expected to rapidly make high-stakes decisions, foster interpersonal relationships, and effectively manage stress in the clinical learning environment of the emergency department (ED), the role EI plays in EM physician success cannot be taken lightly.

Front-line physicians who work in the ED and other acute care settings are at the top the list on physician burnout rates.^1^ Physician suicide continues to be a major challenge for the medical profession, and residents are not spared from this tragedy. EI has been shown to be essential to successfully cope with stress, as well as to create the foundation of sound mental and physical health.^7^^-^^9^ Therefore, the intersection of EI, mental health, and coping abilities underscores the timeliness and importance of better understanding resident EI to promote and inform educational wellness interventions.

No prior studies to date have assessed emotional intelligence exclusively in EM residents, specifically discrepancies in EI across years in training. Recent studies have included investigations of EI in residents of obstetrics and gynecology (OBGYN), orthopedics, otolaryngology, pathology, pediatrics, and general surgery residency programs.^1^^,^^10^^-^^13^ If trends in EM resident trainees do exist, potential training interventions may be appropriately designed to support wellness and address EI. The authors sought to reveal findings that would potentially direct future residency training, curriculum development, and educational programming to prospectively address physician self-awareness and efficacy. If done successfully, such training interventions have the potential to improve wellness, provider-patient communication and, ultimately, health outcomes.

**Table 1:** Emergency medicine residents EI scores

Residents	n	Total EI (Mean ± SD)	95% CI
All	35	103.3 ± 13.5	98.8-107.7
Female	14	106 ± 12	100.0-112.0
Male	21	101 ± 15	95.0-108.0
PGY-1 Residents	12	104 ± 16	95.0-114.0
PGY-2 Residents	11	95 ± 12^*^	88.0-102.0
PGY-3 Residents	12	110 ± 8^*†^	105.0-114.0

*Difference in mean total EI scores between PGY-2 and PGY-3 residents (PGY-2 < PGY-3) was observed to be statistically significant (unpaired t-test, p < 0.01).

†Difference in mean total EI scores between PGY-3 residents and the general population (general population < PGY-3 residents) was observed to be statistically significant (unpaired t-test, p < 0.05).

The aim of the study was to objectively assess the emotional intelligence of a cohort of emergency medicine residents. Specifically, the authors sought to: a) objectively quantify competencies of EI by year of residency training (i.e., first-year residents [or post-graduate year, PGY, 1 residents]; second-year residents [or post-graduate year, PGY, 2 residents]; and third-year residents [or post-graduate year, PGY, 3 residents]); b) identify areas of resident strength and weakness by year of training across EI competency domains; c) determine if there is a statistically significant increase in EI across year of training; and d) identify any trends in emotional intelligence that may exist within each year of residency training.

The authors hypothesized that there are distinct EI competencies with which EM residents may already have mastery of, specifically in stress tolerance, flexibility, impulse control, interpersonal relationships, and empathy, as these are skills that are inherently part of traditional emergency medicine training. The authors also hypothesized that there may be statistically significant increases in composite EI with progressive advancement through residency training. Findings would be applied to inform future curricular development, optimize existing training programs, and create effective wellness interventions for trainees.

## Methods

Study design and participants

This cross-sectional study was conducted at an urban EM residency training program. Study recruitment included all EM residents (i.e., post-graduate years [PGY] 1-3) at Thomas Jefferson University (TJU) Hospital in Philadelphia, Pennsylvania. There were no exclusion criteria for participation in this study. The Department of Emergency Medicine at TJU Hospital sponsors a three-year residency program accredited by the Accreditation Council for Graduate Medical Education (ACGME). There are thirty-six residents enrolled in the program.

EI was measured using a single electronic instrument, the EQ-i 2.0 (described below). Unique log-in accounts to access the instrument via a commercial website were sent to all participants' secure, Institutionally-sponsored e-mail addresses. Residents also completed an electronic consent form, followed by a demographic questionnaire that included their self-reported gender and level of residency training. Surveys were sent to residents in the month of February, which coincides with the middle of the residency academic year.

The study was reviewed by the Institutional Review Board (IRB) of the Sidney Kimmel Medical College of Thomas Jefferson University in Philadelphia, Pennsylvania. After IRB review, the study was deemed compliant with all ethical requirements, and was granted approval by the Board. All participants who completed the assessment did so anonymously; no identifying information was collected. The study did not involve animal or human tissue.

### Study instrument

The EQ-i 2.0 is a psychometrically validated EI assessment tool derived from Bar-On's (1997) conceptual model of emotional intelligence (Multi-Health Systems, 2011).^14^ The EQ-i 2.0 self-assessment is 133 items in length and takes approximately twenty minutes to complete. A participant is asked to read and respond to 133 statements by indicating his/her level of agreement using a 5-point Likert Scale (i.e., 1=never/rarely, 2=occasionally; 3=sometimes; 4=often; 5=always/almost always).^15^

The EQ-i 2.0 uses a normative sample of adults, which includes 4,000 self-report ratings from adults residing in the United States (90% of the sample) and Canada (10% of the sample). The EQ-i is based on data gathered from all 50 U.S. states and the District of Columbia, as well as from all ten Canadian provinces. The normative sample matches Census means and is highly representative of the North American general population.^15^ EQ-i scores are accompanied by respective computed 95% confidence intervals, and built-in correction factors to counter response bias.^15^^,^^16^

The EQ-i 2.0 was chosen for both its validity and reliability. Content validity analyses, exploratory factor analyses, and confirmatory factor analyses suggest that the EQ-i 2.0 is a valid measure of EI. Its validity scales (positive impression, negative impression, and inconsistency index) were also validated through expected differences in scores between known invalid responses and those of control groups.^15^^-^^16^ The EQ-i has sound reliability given its high Cronbach and test-retest values. Its Cronbach's α-value of 0.97 gives this assessment tool a high degree of internal consistency.^16^ Test-retest correlations are also high for EQ-i 2.0 scores at 2-to-4 weeks (r = 0.92) and at 8 weeks (r = 0.81).^16^

### Statistical analyses

Descriptive statistics are used to analyze data. Continuous variables are reported as means with standard deviations. Results were normed to the general North American population, in which a mean EI score is 100 with a standard deviation (SD) of 15. Scores are plotted along a normal distribution, with an average score of 100, an upper limit of 145, and SD of 15. The unpaired, two-sample t-test was used to evaluate differences in means.

Total EI scores for emergency medicine residents were computed by MHS software. Five categorical EI composite scores were calculated, including Self-Perception, Self-Expression, Interpersonal Skills, Decision Making, and Stress Management Composites. Furthermore, 15 subscales of emotional intelligence were generated; these include the following: Self-Regard, Self-Actualization, Emotional Self-Awareness, Emotional Expression, Assertiveness, Independence, Interpersonal Relationships, Empathy, Social Responsibility, Problem Solving, Reality Testing, Impulse Control, Flexibility, Stress Tolerance, and Optimism.

Differences in EI amongst groups varying by gender and PGY-training level were assessed. The t-test was utilized to compare means, with significance defined when p < 0.05. This study was fully reviewed and received exemption status by the Institutional Review Board (IRB) of the Sidney Kimmel Medical College of Thomas Jefferson University in Philadelphia, Pennsylvania.

## Results

Thirty-five EM residents completed the EQ-i 2.0 from a total of 36 eligible residents for a response rate of 97.2%. Total mean EI for resident trainees was 103.3 (95% CI, 99.8-107.7). Table 1 provides a summary of mean EI scores for residents by gender and post-graduate year. Collectively, mean EI were higher in female residents (106; 95% CI, 100-112) than male residents (101; 95% CI, 95-108); the difference in means by gender was not statistically significant.

When examining mean EI scores by year of training, PGY-2s demonstrated the lowest mean EI score (95; 95% CI, 87-104) versus PGY-1s (104, 95% CI, 95-114) and PGY-3s (110, 95% CI, 103-116). Only the difference in means between PGY-3s (110) and PGY-2s (95) was found to be statistically significant (two-sample t-test, p <0.01). When compared to the general population, only the mean difference between PGY-3s (110) and the general population was found to be statistically significant (two-sample t-test, p < 0.05).

**Table 2:** EI composite mean scores of the residency cohort with associated subscale mean scores

EI Composite Scores with Respective Subscale Scores	All Residents (Mean SD)	95% CI
Self-Perception Composite	101 ± 14	96.7-105.9
Self-Actualization	107 ± 13	102.7-111.1
Self-Regard	97 ± 15	91.6-101.6
Emotional Self-Awareness	100 ± 16	94.6-104.9
Self-Expression Composite	99 ± 16	93.1-104.0
Emotional Expression	100 ± 17	94.5-105.7
Assertiveness	97 ± 18	91.5-103.4
Independence	99 ± 15	93.6-103.9
Interpersonal Composite	106 ± 12	102.2-110.4
Interpersonal Relationships	105 ± 11	101.7-109.3
Empathy	107 ± 12	102.9-111.0
Social Responsibility	103 ± 14	98.8-108.0
Decision Making Composite	104 ± 13	99.2-108.0
Reality Testing	103 ± 15	97.8-107.4
Problem Solving	100 ± 12	96.0-104.0
Impulse Control	106 ± 16	100.6-111.3
Stress Management Composite	104 ± 14	99.5-108.8
Flexibility	102 ± 15	96.8-106.8
Stress Tolerance	106 ± 13	101.3-110.1
Optimism	103 ± 15	97.7-107.4

Table 2 provides a summary of mean composite EI scores for the entire residency cohort, along with the three-associated subscale mean scores for each EI composite. The highest composite scores were in interpersonal skills (106; 95% CI, 100-108) and stress management (104; 95% CI, 101-109). The lowest composite score for the residency cohort, which was also below the general population mean, was self-expression (98.6; 95% CI, 93-104). Residents scored higher than the general population across all remaining composite categories. With regards to specific EI subscale domains (Table 2), the EM residency cohort demonstrated strengths in the following areas: self-actualization (107); empathy (107); impulse control (106); stress tolerance (106), and interpersonal relationships (105). The lowest subscale scores across all residents were observed in assertiveness (97) and self-regard (97). When the five composite EI scores are examined across year of training (Table 3), PGY-2s consistently scored lower than their PGY-1 and PGY-3 counterparts. Statistically significant differences in means were observed between PGY-2s and PGY-3s, specifically in stress management (two-sample t-test, p <0.05); self-perception (p <0.01); and self-expression (p <0.01).

**Table 3:** EI composite and subscale mean scores by level of training (PGY1-3)

EI Composite Scores with Respective Subscale Scores	PGY-1		PGY-2		PGY-3	
	Mean, SD	95% CI	Mean, SD	95% CI	Mean, SD	95% CI
Self-Perception Composite	104 ± 18	94-114	93 ± 12^*^	86-100	106 ± 9.0^*^	101-111
Self-Actualization	110 ± 13	102-117	99 ± 15†	91-108	111 ± 11†‡	105-117
Self-Regard	100 ± 17	90-109	89 ± 11¶†	82-95	101 ± 8.4†	96-106
Emotional Self-Awareness	100 ± 21	88-113	95 ± 9.0	89-100	104 ± 12	97-111
Self-Expression Composite	100 ± 21	87-112	90 ± 17¶*	80-100	106 ± 8.4^*^	101-110
Emotional Expression	100 ± 10	95-106	96 ± 10	90-102	103 ± 13	96-110
Assertiveness	99 ± 16	90-108	88 ± 15 ¶†	79-96	105 ± 7.3†	101-109
Independence	99 ± 14	91-107	90 ± 14 ¶†	82-98	107 ± 13†	99-114
Interpersonal Composite	109 ± 12	102-116	100 ± 18	90-111	110 ± 7.7§	105-114
Interpersonal Relationships	105 ± 19	95-116	103 ± 15	94-112	108 ± 7.4	104-112
Empathy	110 ± 16	100-118	102 ± 17	92-111	109 ± 17‡	100-119
Social Responsibility	107 ± 14	99-115	95 ± 13†	87-102	108 ± 8.7†	103-113
Decision Making Composite	101 ±18	91-111	99 ± 15	90-108	110 ± 14	102-118
Reality Testing	102 ± 12	95-108	98 ± 21	86-110	108 ± 10	102-114
Problem Solving	97 ± 18	87-108	94 ± 9.0†	89-100	108 ± 9.2†	103-113
Impulse Control	104 ± 13	96-112	106 ± 12	90-113	108 ± 13	100-116
Stress Management Composite	105 ± 19	94-116	97 ± 15^*^	88-106	110 ± 12*§	103-117
Flexibility	103 ± 14	95-111	97 ± 13	89-104	105 ± 14	98-113
Stress Tolerance	103 ± 13	96-111	101 ± 13†	93-109	113 ± 7.6†‡	109-117
Optimism	105 ± 16	96-115	96 ± 14†	88-104	106 ± 8.6†	101-110
Total EI	104 ± 16	95-114	95 ± 14	87-104	110 ± 12	103-116

*Differences in specific mean composite EI scores between PGY-2 and PGY-3 residents (where PGY-2 < PGY-3) were observed to be statistically significant for self-perception (unpaired t-test, p <0.01), self-expression (unpaired t-test, p < 0.01), and stress management (unpaired t-test, p < 0.05).

†Differences in specific mean subscale EI scores between PGY-2 and PGY-3 residents (where PGY-2 < PGY-3) were observed to be statistically significant for self-actualization (unpaired t-test, p < 0.05), self-regard (unpaired t-test, p < 0.01), assertiveness (unpaired t-test, p < 0.01), independence (unpaired t-test, p < 0.01), social responsibility (unpaired t-test, p < 0.01), problem solving (unpaired t-test, p < 0.01), stress tolerance (unpaired t-test, p < 0.05), and optimism (unpaired t-test, p < 0.05).

‡Differences in specific mean subscale EI scores between PGY-3 residents and the general population (where general population < PGY-3 residents) were observed to be statistically significant for self-actualization (unpaired t-test, p < 0.05), empathy (unpaired t-test, p < 0.05), and stress tolerance (unpaired t-test, p < 0.01).

¶Differences in specific mean composite and mean subscale EI scores between PGY-2 residents and the general population (where PGY-2 residents < general population) were observed to be statistically significant for self-expression (unpaired t-test, p < 0.05), self-regard (unpaired t-test, p < 0.05), assertiveness (unpaired t-test, p < 0.01), and independence (unpaired t-test, p < 0.05).

§Differences in specific mean composite EI scores between PGY-3 residents and the general population (where general population < PGY-3 residents) were observed to be statistically significant for interpersonal skills (unpaired t-test, p < 0.05) and stress management (unpaired t-test, p < 0.05).

 PGY-1 subgroup analysis (Table 3) revealed strengths in empathy (110), self-actualization (110), social responsibility (107), optimism (105), interpersonal relationships (105), and impulse control (104); lowest PGY-1 scores were observed in problem-solving (97), assertiveness (99), and independence (99).

PGY-2 subgroup analysis (Table 3) revealed lower scores in self-regard (89), assertiveness (88), independence (90), problem-solving (94), flexibility (97), and optimism (96). With the exception of impulse control, PGY-2s scored lower across all subscales of EI when compared to their PGY-1 and PGY-3 counterparts. Statistically significant differences were noted in PGY-2 subscale scores when compared to the general population, specifically for self-regard (two-sample t-test, p <0.05) and two of the three sub-scales within the self-expression composite: assertiveness (two-sample t-test, p <0.01); and independence (p <0.05).

PGY-3 residents scored highest in nearly all (14 out of 15) subscales of EI (Table 3). Highest PGY-3 EI scores were in stress tolerance (113), self-actualization (111), and empathy (109). All PGY-3 mean scores of EI by subcategory were above the means for the general population, but were only statistically significant for stress tolerance (two-sample t-test, p <0.01); self-actualization (p <0.05); and empathy (p <0.05). Relative to their EM peers, statistically significant differences in EI subscale scores were observed between PGY-2s and PGY-3s for self-actualization (two-sample t-test, p <0.05), self-regard (p <0.01), assertiveness (p <0.01), independence (p <0.01), social responsibility (p <0.01), problem solving (p <0.01), stress tolerance (p <0.05), and optimism (p <0.05).

## Discussion

In this study, patterns of emotional intelligence in EM residents are examined across gender and post-graduate year of training. To this effect, a working EI profile of EM residents at our institution is defined, and trends across our three progressive classes of residents are described. Residents scored above the general population in total EI and across four of the five composite scales. A positive linear relationship between years of training and EI was not observed in the sample. In fact, the mean PGY-2 EI score was not only lower than the mean PGY-1 score but also below the national average. This difference in total EI between PGY-2s and the general population was not statistically significant. A statistical significant difference in total EI score, however, was observed between PGY-2 and PGY-3 cohorts, raising the question as to what are the factors that contributed to this finding.

Two potential possibilities merit consideration: either the PGY-2 cohort was an outlier, with poor baseline EI; or being in the middle of their training, when stress and self-doubt are at their highest, was responsible for their underperformance. The second-year EM resident is expected to evaluate a larger volume of patients; to have acquired a higher level of medical knowledge, and to care for critically-ill patients with a marked increase in responsibility.^17^ The literature suggests that caring for severely- and critically-ill patients can have a negative toll on a provider's emotional and physical well-being, especially in the nascent stages of transition.^18^^-^^20^ Further investigation with longitudinal reassessment of trainee EI may be helpful in clarifying this observation. If so, additional wellness interventions may be required at this potential training nadir.

Based on overall findings, the data suggests that residents may be struggling with self-expression and self-perception. This is perhaps not surprising when one considers the overall milieu of healthcare-based education, which has been criticized for poor adoption of the medical humanities,^21^ a meagre emphasis on self-expression,^22^ and questionable self-reflective exercises.^23^ Training environments across residencies are quite variable, and often dependent on the supervising faculty to create a culture that encourages reflection and feedback. While some supervising faculty physicians may nurture environments that support discussion and debate, others may opt for a different, less supportive style.^24^ Lastly, it is plausible that residents may struggle with their large and varied time commitments. Self-expression is difficult to practice when balanced against the clinical, academic, and professional demands a trainee requires to advance.

As stewards of physicians-in-training, residency leadership have the opportunity to create training opportunities to assist the resiliency needed to cope with these challenges. Two particular areas of interest that can potentially address the aforementioned include learner well-being and resident self-expression.

Training interventions that promote resident wellness are paramount,^24^ and can benefit from an improved understanding of cohort EI strengths and weakness. One the most studied interventions on resident wellness, thus far, has been the introduction of work hour restrictions for residents, with many studies revealing a positive impact on self-perceived resident wellness, fatigue, burnout, and physiological distress.^25^^-^^27^ Stanford University provides another exemplar, their Balance in Life Program, which provides their residents with mentorship and leadership training; stocks resident environments with healthy foods and snacks; offers residents resources that foster mental, emotional and physical health; and hosts social gatherings and events.^28^

Medical education programs that encourage self-expression exist and typically approach learners through various artistic activities such as self-reflective exercises.^29^^-^^32^ They have shown modest improvements in medical student EM clerkship performance.^32^

The fact that second-year residents exhibited lower scores relative to the general population in self-regard and assertiveness, as well as lower scores in stress tolerance and optimism relative to their counterparts, is of high concern. Transitions in residency training, graded responsibility, and increased workloads are tipping points in residency, and require sound emotional intelligence to counteract them. Attention to trainee stress tolerance, assertiveness, and optimism is key to promoting a culture of wellness and resiliency during post-graduate training. In addition to the aforementioned, promoting an open culture of learning and discussion in the emergency department, strengthening resident education, and encouraging real-time feedback for residents can further promote resident wellness.

Integrating newer learning modalities, such as simulation-based education,^33^ mixed modality/asynchronous learning,^34^^-^^36^ and the free-open-access-medical education (FOAMED) electronic movement^36^ can also foster the intellectual exchange of ideas and socialize the learning experience.^35^ Additionally, placing an emphasis on continuous, high-quality feedback in the ED can potentially promote EI through better and more accurate reflection.^36^

That PGY-3s demonstrated the highest EI scores is encouraging. While correlation is not causation, this potentially suggests that EM residency training may have a positive impact on the development of emotional intelligence. While intuitive, other specialties have demonstrated the opposite pattern.^11^ Given the marked difference comparing surgical subspecialty training to EM, it's unclear if the contradictory results are related to practice variation or represent a true dispute. This contradiction merits further study employing qualitative approaches (i.e., resident interviews, focus group) and perhaps aptitude-based assessments of emotional intelligence.

### Limitations

Although our study is one of the first to measure EI in EM residents, it has a number of limitations. Despite a >97% response rate, our total cohort size was 35 residents or 11-12 per post-graduate training year. A larger sampling of participants would have provided for more robust data and trends of emotional intelligence across years of training. Furthermore, our study takes a 'point-in-time' view of EI with no assessment of how EI trends for an individual over time. Subsequent studies of emotional intelligence should re-test participants at the beginning and start of each year of residency training; in this manner, each participant would also serve as a control for himself/herself. Similarly, the data was not segregated by age.

The science of assessing EI remains a challenge, and despite being a psychometrically validated tool, the EQ-i 2.0 has its own intrinsic limitations. The EQ-i 2.0 relies on a participant's self-assessment. While the instrument incorporates correction factors for item responses, it is conceivable that participants may opt to deliberately overestimate and/or underestimate their responses to statements in hopes of improving their EI scores. While this would be difficult to account for with the EQ-i 2.0, other instruments of emotional intelligence are aptitude- and task-based, making deliberate over- and underestimations more challenging. The Mayer-Salovey-Caruso Emotional Intelligence Test (MSCEIT), as an example, is an ability-based measure of emotional intelligence, and tests the participant's ability to perceive, use, understand, and regulate his/her emotions.^38^ Future studies of emotional intelligence utilizing several different assessments and correlating results may offer a more accurate representation of EI, and change in EI, over the course of training.

Finally, the current study does not correlate EI with resident performance during training. It would be of value to ascertain if EI has the ability to predict resident performance (i.e., does a low subscale score in empathy correlate with poor patient satisfaction ratings). Similarly, future studies should also aim to collect data on residents' perceived stress level. If a correlation between EI and perceived stress level does exist, it is quite possible that EI assessments have the potential to identify vulnerable and at-risk resident physicians who may require immediate and targeted wellness interventions.

## Conclusions

The present study identifies EI strengths and weaknesses in a cohort of EM residents, which has the potential to inform education leadership in efforts to improve wellness and training. A linear relationship of emotional intelligence with EM residency training was not identified in the current study; however, data suggests that there are specific aspects of EI that decline in the second year of training. While a qualitative evaluation is needed to identify the underlying themes that would explain these observations, residency and wellness training interventions should address learner self-regard, assertiveness, stress tolerance, and self-expression. Findings of this study offer significant insights into effective medical education programming in EM, specifically for program design for EM resident trainees, particularly during a climate of heightened awareness of resident burnout, mental health, resiliency, and wellness. The overarching goal will be to align training in EI with core competencies and milestones set forward by the Accreditation Council for Graduate Medical Education. Further research will be needed to clarify the natural course of EI development in resident learners and its impact on success during training.

### Conflict of Interest

The authors declare that they have no conflict of interest.
